# A Case of Ascending Colon Stricture Following Appendicitis with Abscess Formation

**DOI:** 10.7759/cureus.110564

**Published:** 2026-06-09

**Authors:** Takuji Ota, Junichi Goto, Kengo Kita, Masanori Sato, Masahiro Hattori, Hiroaki Takahashi

**Affiliations:** 1 Department of Surgery, Sapporo Hokuyu Hospital, Sapporo, JPN

**Keywords:** appendicitis, colonic stricture, complicated appendicitis, hemodialysis, interval appendectomy, laparoscopic surgery

## Abstract

Colonic stricture secondary to appendicitis is exceptionally rare. We report a unique case of ascending colon stricture developing one year after treatment for complicated appendicitis in a patient on long-term hemodialysis. A 68-year-old male with a 26-year history of hemodialysis underwent percutaneous drainage for appendicitis with abscess formation, followed by an interval laparoscopic appendectomy. One year postoperatively, he developed nausea and vomiting. Colonoscopy revealed a circumferential stricture in the ascending colon without mucosal abnormalities. A contrast enema demonstrated a 2 cm apple-core stricture. Laparoscopic right colectomy was successfully performed despite extensive adhesions. Histopathological examination confirmed chronic inflammatory stricture with submucosal fibrosis and no evidence of malignancy. The patient recovered well with the resolution of symptoms. This case demonstrates that complicated appendicitis with abscess formation can cause delayed colonic stricture, particularly in immunocompromised patients such as those on chronic hemodialysis. Clinicians should be aware of this potential delayed complication when managing patients with a history of complicated appendicitis.

## Introduction

Colonic strictures are most commonly caused by malignant tumors; however, benign strictures can result from various conditions, including diverticulitis, inflammatory bowel disease, ischemic colitis, radiation-induced colitis, and endometriosis [1,2]. Complicated appendicitis with perforation or abscess formation can cause inflammatory changes in adjacent organs, and colonic stricture secondary to appendicitis is exceptionally rare, with most reported cases involving the sigmoid colon. Patients on chronic hemodialysis may be predisposed to excessive fibrosis owing to altered inflammatory responses and impaired wound healing [3,4]. We report a rare case of ascending colon stricture that developed one year after an interval laparoscopic appendectomy for complicated appendicitis in a long-term hemodialysis patient successfully treated with laparoscopic right colectomy.

## Case presentation

A 68-year-old male presented with nausea and vomiting. His medical history included chronic renal failure requiring hemodialysis for 26 years and lower limb arterial occlusive disease. One year and eight months before the current presentation, he developed a fever and right lower quadrant abdominal pain. Investigation revealed acute appendicitis with abscess formation (Figure 1).

**Figure 1:**
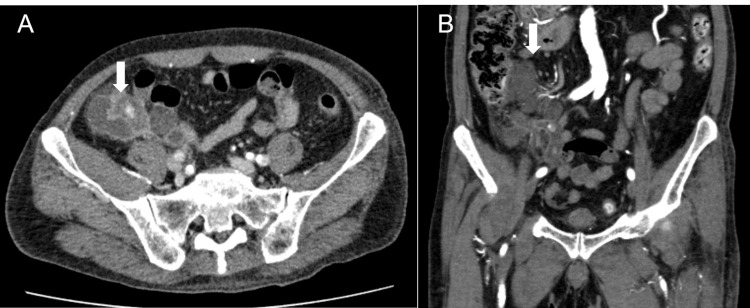
Contrast-enhanced CT at the initial presentation Axial (A) and sagittal (B) views of the enlarged appendix and a pericecal/peri-ascending colonic abscess (white arrow). These findings were consistent with acute appendicitis with abscess formation.

He was admitted for treatment. Nonoperative management with intravenous antibiotics failed, necessitating percutaneous abscess drainage under ultrasound guidance (Figure 2).

**Figure 2:**
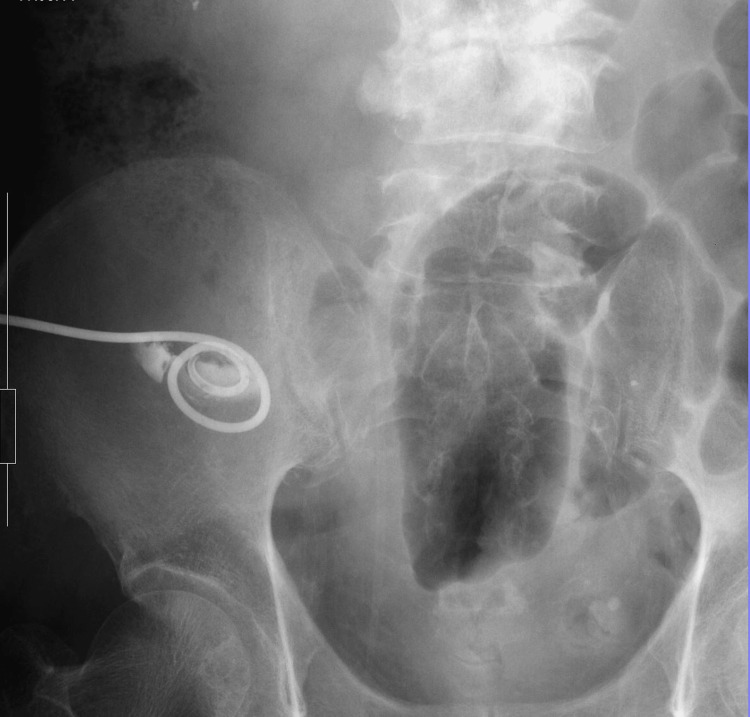
Image obtained during percutaneous abscess drainage Under ultrasound guidance, a drainage catheter was placed within the abscess cavity.

Contrast imaging performed during drainage demonstrated no enteric fistula between the colonic lumen and the abscess cavity. The drainage led to prompt normalization of inflammatory markers. Eight months after the initial admission, a total colonoscopy was performed before the interval appendectomy to exclude underlying neoplastic lesions, and no mucosal abnormalities were identified. The interval laparoscopic appendectomy was planned as definitive treatment given the risk of recurrent appendicitis and the patient's overall clinical background. The procedure was performed using a three-port technique. Intraoperatively, the appendix was found in a pre-ileal position, and mild periappendiceal inflammation was observed, accompanied by loose adhesions (Figure 3).

**Figure 3:**
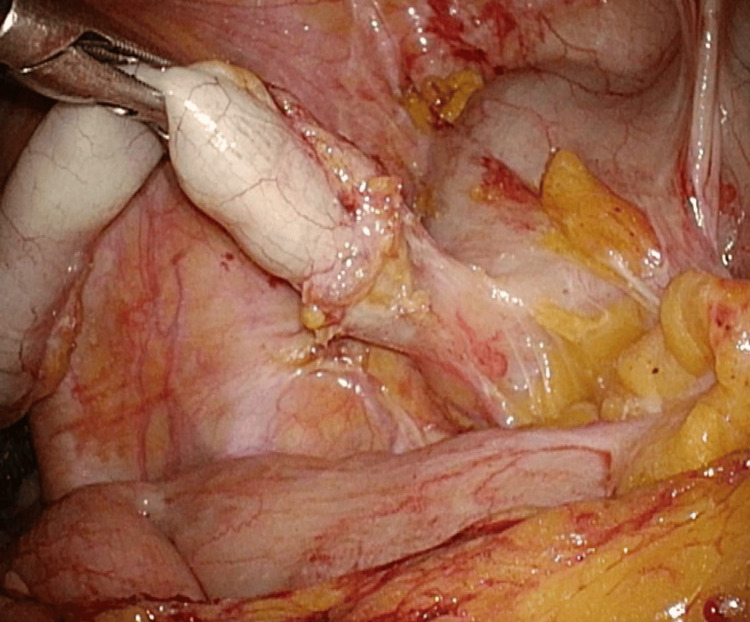
Intraoperative view from the initial laparoscopic appendectomy Abscess formation had resolved, and although periappendiceal inflammatory changes remained, laparoscopic appendectomy was performed without technical difficulty.

Appendectomy was performed without technical difficulty. The appendiceal stump was doubly ligated using non-absorbable sutures. The operative time was one hour with minimal blood loss. The postoperative course was uneventful, and the patient was transferred to a rehabilitation facility.

One year after the interval laparoscopic appendectomy, the patient developed recurrent nausea and vomiting and was referred back to our department. Physical examination revealed a height of 158.0 cm and a weight of 51.8 kg; the abdomen was flat and soft, with a surgical scar at the umbilicus. Laboratory investigations demonstrated anemia (hemoglobin 7.4 g/dL; reference range: 13.5-17.1 g/dL) and hyponatremia (sodium 127 mmol/L; reference range: 136-145 mmol/L), with no elevation in white blood cell count or C-reactive protein. Tumor markers, including CEA and CA19-9, were within normal range.

On the second admission, colonoscopy revealed a circumferential stricture in the ascending colon through which the colonoscope could not be passed. No mucosal elevated lesions were identified (Figure 4A). Contrast enema demonstrated an approximately 2 cm-long stricture (Figure 4B).

**Figure 4:**
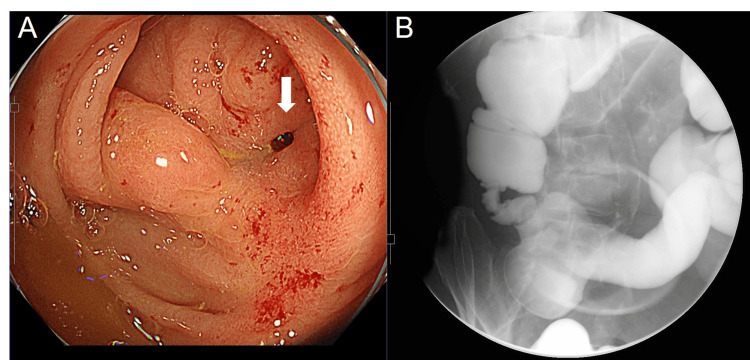
Colonoscopy and contrast enema findings A: A circumferential stricture was observed in the ascending colon (white arrow) during the colonoscopy. No mucosal elevated lesion was identified; however, passage of the colonoscope through the stenosis was not possible. B: A 2 cm long stenotic segment was demonstrated during the contrast enema. These findings confirmed the diagnosis of benign ascending colon stricture.

Based on these findings, a diagnosis of bowel obstruction due to ascending colon stricture was established, and surgical intervention was planned. Although malignancy could not be entirely excluded based on imaging alone, surgical intervention was planned for both therapeutic and diagnostic purposes. Laparoscopic surgery was initiated using a five-port technique. Intraoperative examination revealed fibrotic thickening of the ascending colon (Figure 5).

**Figure 5:**
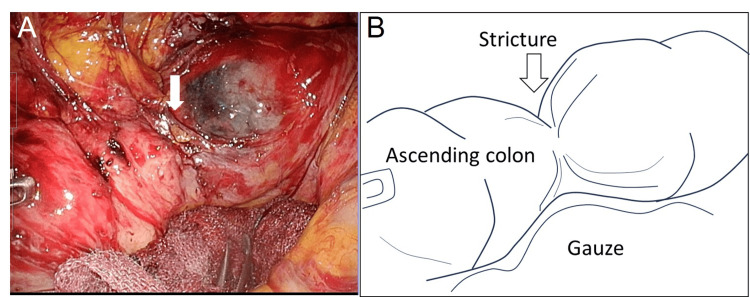
Intraoperative findings and schematic illustration of laparoscopic right colectomy Fibrotic changes and a stenotic segment were observed on the serosal surface of the ascending colon (arrow). The schematic illustration was created by the authors using Microsoft PowerPoint (Microsoft Corporation, Redmond, WA, USA).

Although the surrounding tissues showed fibrotic changes, laparoscopic adhesiolysis was feasible. Right colon mobilization was performed, and the strictured segment was exteriorized through a small skin incision. Functional end-to-end anastomosis was performed using a 60-mm linear stapler. The operative time was five hours with minimal blood loss.

The postoperative course was favorable. Oral intake was resumed on postoperative day three, and the preoperative symptoms resolved without complications. Due to the time required for improvement in functional independence, the patient was transferred to another hospital for rehabilitation on postoperative day 57.

Histopathological examination revealed no evidence of acute inflammation or malignancy. Fibrotic thickening and small vessel proliferation were predominantly observed in the submucosal layer, confirming the diagnosis of colonic stricture due to chronic inflammation (Figure 6).

**Figure 6:**
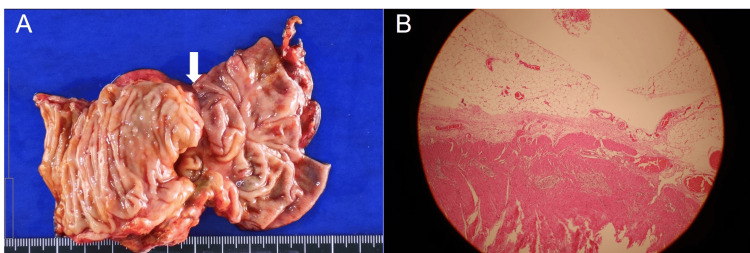
Histopathological findings A: Resected specimen with a 2 cm circumferential stricture (white arrow); (B) Hematoxylin and eosin (H&E)-stained section (×40) shows the stenotic area with fibrotic thickening of the submucosa and features consistent with chronic inflammation. These histopathological findings confirmed the diagnosis of chronic inflammatory stricture, excluding malignancy.

## Discussion

Benign colonic strictures are broadly classified into those originating from the colon itself and those resulting from involvement of adjacent organs. The former includes post-traumatic scarring, inflammatory bowel diseases such as Crohn's disease and ulcerative colitis, infections, complicated diverticulitis, and postoperative anastomotic strictures. Strictures due to inflammatory involvement from other organs include endometriosis, pancreatitis, and mesenteric panniculitis. Diverticular disease is a leading cause of benign colonic strictures; stricture formation is thought to result from recurrent acute inflammation and progressive fibrosis of the colonic wall [1,2]. In the present case, although colonic diverticula were present, there was no evidence of diverticulitis on imaging. A literature search was conducted using the keywords 'appendicitis,' 'abscess,' and 'intestinal stricture.' To the best of our knowledge, there are only five detailed cases of intestinal stricture following appendicitis, including our own (Table 1).

**Table 1:** Reported cases of intestinal stricture following appendicitis

Author, year, and reference no.	Age	Sex	Chief complaint	Stricture location	Treatment	Diagnosis
Ishikawa et al., 1992 [5]	42	Female	Lower abdominal pain	Sigmoid colon	Appendectomy and sigmoid colectomy	Perforated appendicitis
Tasker AD et al., 1995 [6]	49	Male	Diarrhea	Small intestine	Appendectomy and limited small bowel resection	Abscess-forming appendicitis
Tasker AD et al., 1995 [6]	54	Male	Central abdominal pain	Sigmoid colon	Appendectomy	Abscess-forming appendicitis
Yamamoto et al., 2003 [7]	30	Male	Constipation	Sigmoid colon and ureter	Appendectomy and sigmoid colostomy	Perforated appendicitis
Our case	68	Male	Vomitting	Ascending colon	Right colectomy	Post-appendectomy for abscess-forming appendicitis

Four of the patients from these case reports were male, and one was female. The stricture was located in the sigmoid colon in two cases, the sigmoid colon and ureter in one case, the small intestine in one case, and the ascending colon in one case. The reported intestinal strictures involved the sigmoid colon and small intestine. The spread of inflammation from the appendix to adjacent intestinal segments is thought to depend on the anatomical position of the appendix and the extent of abscess formation. Notably, one of the reported cases was resolved successfully with nonoperative management.

Three distinctive features characterize this case: a chronic course following appendicitis with abscess formation, severe stricture requiring surgical intervention, and occurrence in a hemodialysis patient. Abscess formation in appendicitis indicates substantial inflammatory involvement of surrounding tissues. Increased inflammatory cell infiltration and cytokine production around the abscess may have contributed to matrix metalloproteinase-9 (MMP-9) activation and tissue remodeling, thereby promoting fibrosis and potentially leading to stricture formation [8-10].

In hemodialysis patients, tissue repair may be impaired owing to sustained elevation of inflammatory cytokines from chronic inflammation, hypoalbuminemia from malnutrition, and deficiency of trace elements important for wound healing [3,4]. These factors may have promoted excessive fibrosis following appendicitis in the present patient.

While post-inflammatory fibrosis is considered the most likely mechanism in our case, alternative etiologies warrant consideration. Ischemic stricture is a plausible alternative etiology in this patient, given his 26-year history of hemodialysis and peripheral arterial occlusive disease. However, the clinical course showed no signs of intestinal ischemia, and the location of the stricture corresponded closely to the site of the prior abscess. Furthermore, histopathological examination demonstrated submucosal fibrotic thickening consistent with chronic inflammation, with no features suggestive of ischemic injury. These findings support post-inflammatory fibrosis as the most likely underlying mechanism, although ischemic contribution cannot be entirely excluded.

Regarding treatment selection, endoscopic balloon dilatation is an option for benign colonic strictures. However, surgical treatment was selected in this case because the stricture was severe with incomplete endoscopic passage, and hemodialysis patients have impaired wound healing, increasing the risk of perforation from endoscopic procedures. In patients undergoing hemodialysis, laparoscopic colorectal surgery appears to be as safe as open surgery and may facilitate earlier resumption of oral intake, consistent with the benefits of minimally invasive approaches [11]. Despite the anticipated severe adhesions, we successfully avoided unnecessary bleeding and tissue injury by utilizing optimal laparoscopic visualization and proceeding with the appropriate dissection planes.

## Conclusions

In this study, we present a case of colonic stricture that occurred following surgical intervention for complicated appendicitis in a patient undergoing chronic kidney dialysis. Complicated appendicitis may result in inflammatory involvement of adjacent intestinal segments. When intestinal stricture develops in a delayed manner, alternative etiologies, particularly malignancy and ischemic colitis, must be systematically excluded before determining the appropriate treatment strategy.
